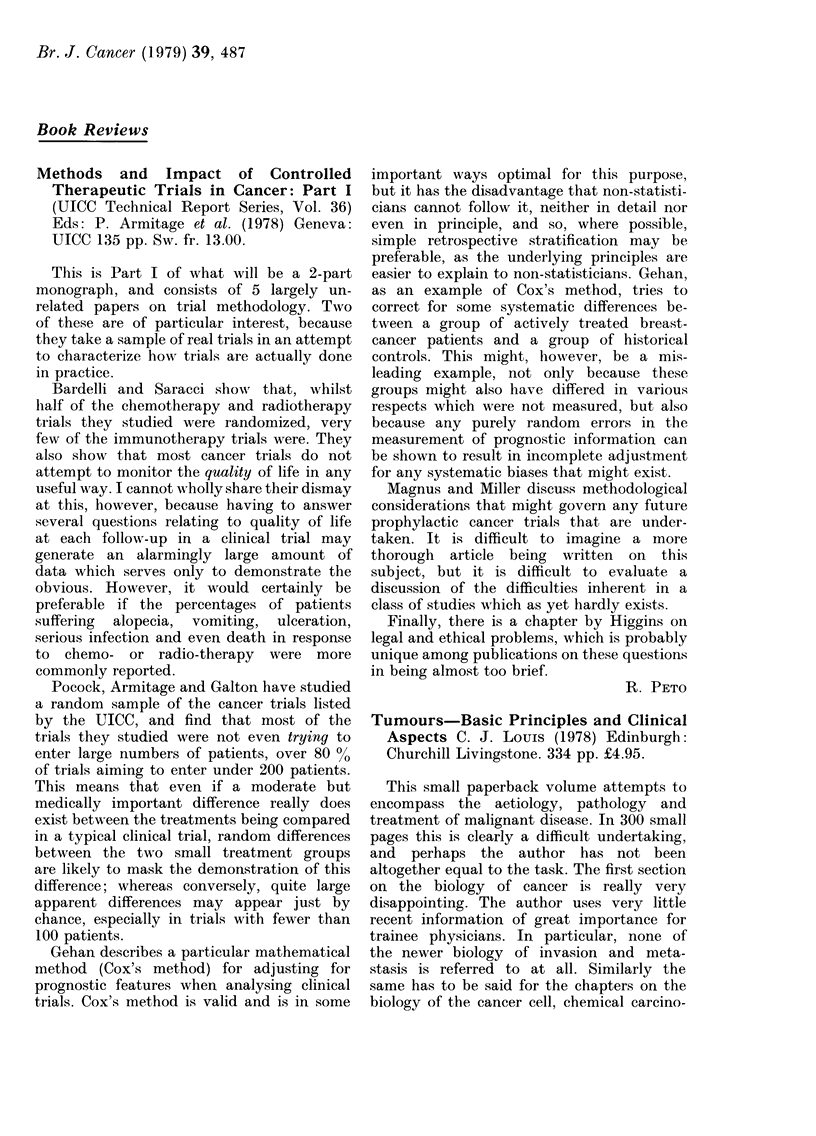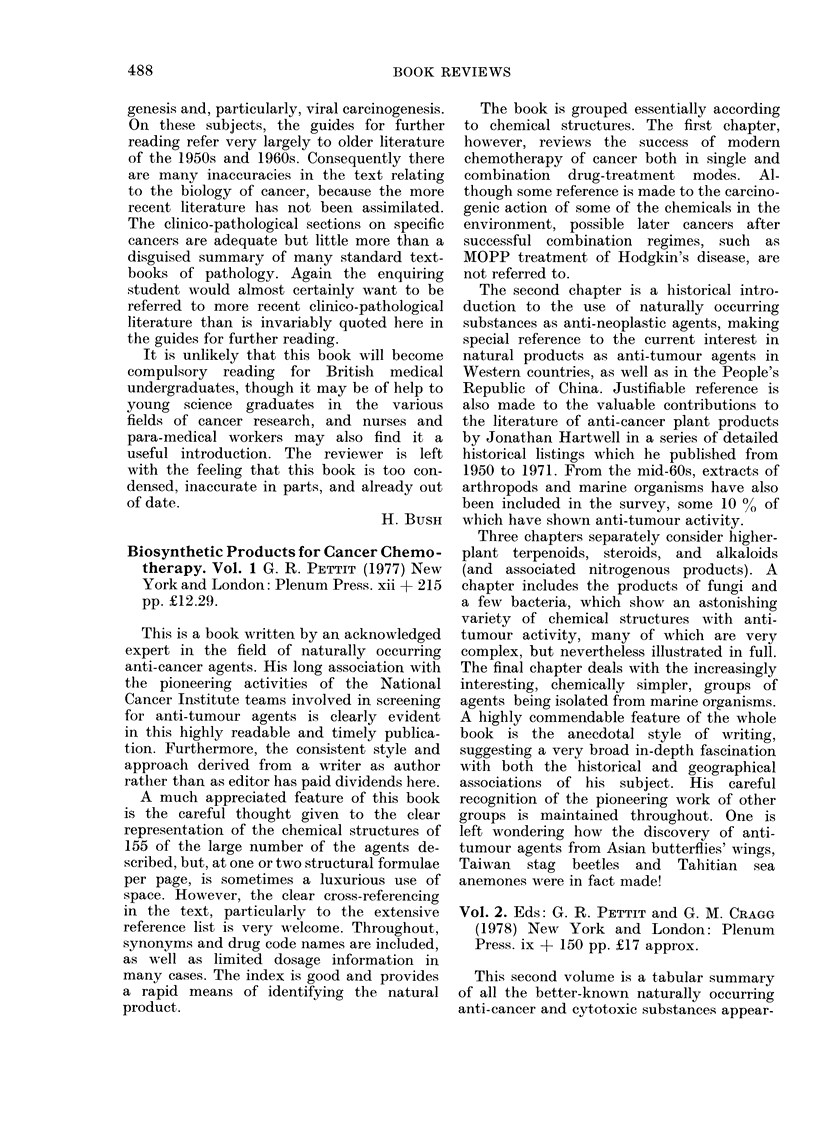# Tumours—Basic Principles and Clinical Aspects

**Published:** 1979-04

**Authors:** H. Bush


**Tumours—Basic Principles and Clinical Aspects** C. J. Louis (1978) Edinburgh: Churchill Livingstone. 334 pp. £4.95.

This small paperback volume attempts to encompass the aetiology, pathology and treatment of malignant disease. In 300 small pages this is clearly a difficult undertaking, and perhaps the author has not been altogether equal to the task. The first section on the biology of cancer is really very disappointing. The author uses very little recent information of great importance for trainee physicians. In particular, none of the newer biology of invasion and metastasis is referred to at all. Similarly the same has to be said for the chapters on the biology of the cancer cell, chemical carcinogenesis and, particularly, viral carcinogenesis. On these subjects, the guides for further reading refer very largely to older literature of the 1950s and 1960s. Consequently there are many inaccuracies in the text relating to the biology of cancer, because the more recent literature has not been assimilated. The clinico-pathological sections on specific cancers are adequate but little more than a disguised summary of many standard textbooks of pathology. Again the enquiring student would almost certainly want to be referred to more recent clinico-pathological literature than is invariably quoted here in the guides for further reading.

It is unlikely that this book will become compulsory reading for British medical undergraduates, though it may be of help to young science graduates in the various fields of cancer research, and nurses and para-medical workers may also find it a useful introduction. The reviewer is left with the feeling that this book is too condensed, inaccurate in parts, and already out of date.

H. BUSH